# Predicting recreational therapy engagement in veterans’ long-term care: a machine learning approach

**DOI:** 10.3389/frhs.2026.1735411

**Published:** 2026-04-30

**Authors:** Mohammad Najeh Samara, Kimberly D. Harry, Brittany Weissman, Kara V. Dopp

**Affiliations:** 1 School of Systems Science and Industrial Engineering, Binghamton University, Binghamton, NY, United States; 2 New York State Veterans Home at Oxford, Oxford, NY, United States

**Keywords:** activity preferences, class imbalance, long-term care, machine learning, participation prediction, predictive modeling, Random Forest, recreational therapy

## Abstract

**Background:**

There is limited understanding regarding the factors that predict recreational therapy engagement among veterans in long-term care facilities. We aimed to develop and validate machine learning models to predict recreational therapy participation and identify key factors influencing engagement patterns among veterans in long-term care facilities.

**Methods:**

In this cross-sectional observational study, we used data from 57 veterans aged 18 years and above at the New York State Veterans Home at Oxford. Data were collected through a comprehensive self-administered survey capturing demographic characteristics, participation patterns, and activity preferences. Two binary outcome variables were constructed: high participation and any participation. Five machine learning algorithms (Random Forest, Decision Tree, Gradient Boosting, and Logistic Regression with L1 and L2 regularization) were systematically evaluated using Leave-One-Out Cross-Validation for high participation and 5-fold Stratified Cross-Validation for any participation. Feature selection was implemented using SelectKBest with f_classif scoring, and class imbalance was addressed through balanced weighting techniques.

**Results:**

Random Forest emerged as the optimal algorithm for both prediction tasks, achieving F1-scores of 0.860 ± 0.347 for high participation prediction and balanced accuracy of 0.619 ± 0.081 for any participation prediction. Feature importance analysis revealed activity preference diversity (Gini importance: 0.293) and total preferences (0.254) as the primary predictors of high participation, while facility tenure (0.268) was the strongest predictor of any participation. Veterans with preference diversity >4.5 activities combined with satisfaction scores >3.84 achieved 100% observed probability of sustained high participation [*n* = 5; 95% exact binomial CI: (47.8%, 100%)], though this estimate should be interpreted cautiously given the small subgroup size. New residents (≤1.5 years) with limited preferences demonstrated the highest risk for non-participation. Group activities (Gini importance: 0.143) and spiritual activities (Gini importance: 0.100–0.101) emerged as significant predictors across both models.

**Conclusions:**

This research provides the first proof-of-concept demonstration of a machine learning approach for predicting recreational therapy engagement among veterans in long-term care facilities, establishing methodological feasibility and generating testable hypotheses for prospective multi-site validation. Activity preference diversity and facility tenure serve as primary determinants of participation, with a critical 1.5-year adaptation period identified for intervention targeting. These predictive models can be applied during admission or early in residency to identify veterans at risk of low participation, enabling recreational therapy staff to implement tailored, proactive engagement strategies before disengagement occurs.

## 1 Introduction

### 1.1 Background

Veterans residing in long-term care (LTC) facilities represent a rapidly growing population with unique therapeutic needs. Compared to the general LTC population, veterans have a higher prevalence of service-related physical disabilities, chronic pain, traumatic brain injury, and mental health conditions such as post-traumatic stress disorder (PTSD) and moral injury (1). They also often share distinct cultural experiences and social identities shaped by military service, which influence their therapeutic engagement and activity preferences (2). In 2013, 47.3% of U.S. veterans were aged 65 or older, with projections indicating over 5 million veterans aged 75 and above by 2023 (3, 4). This significant demographic shift underscores the urgent need for specialized interventions that address the evolving care requirements of aging veteran populations. In response to these demographic changes, recreational therapy (RT) has increasingly been recognized as a critical component of comprehensive veteran care. RT programs offer proven therapeutic benefits that support physical, emotional, and social well-being among older adults in institutional settings (5, 6). Current evidence demonstrates that well-designed RT interventions significantly improve multiple health outcomes, with programs incorporating tai chi, walking, dancing, and therapeutic activities showing measurable improvements in mobility, balance, and functional performance (7).

Beyond physical benefits, recreational therapy has proven effective in addressing psychological and social needs among long-term care residents. Systematic reviews demonstrate significant improvements in depression symptoms following RT interventions, while technologically-mediated recreational programming has been associated with increased quality of life (8). Group-based therapeutic activities have shown particular promise in enhancing sociality and social relationships among elderly residents, addressing the critical issue of social isolation in institutional care settings (9). However, engagement in RT varies considerably among residents and is influenced by complex interactions between multiple factors. Social support from staff and peers plays a significant role in enhancing participation and satisfaction, while the perceived benefit of activities strongly influences veteran motivation to engage (10, 11). Demographic characteristics such as age, gender, and military experience further shape how veterans interact with RT programming, with older veterans often favoring structured indoor programs while younger cohorts may prefer outdoor activities (12, 13). Moreover, satisfaction emerges as both a critical outcome measure and a powerful predictor of sustained participation in therapeutic programs. Research demonstrates that satisfaction with leisure activities is closely linked to psychological well-being, with diverse and continuous engagement leading to higher life satisfaction among elderly populations (14). The multidimensional nature of satisfaction encompasses psychological, educational, and physiological components that collectively influence continued participation in RT programs (15, 16).

Given the complexity of factors influencing RT engagement and the critical importance of sustained participation for therapeutic outcomes, traditional clinical approaches relying solely on professional judgment may fail to consistently identify veterans at risk for low participation. The multifactorial nature of engagement—involving demographic characteristics, activity preferences, satisfaction levels, and social support—creates challenges for clinical staff attempting to predict participation patterns and implement timely interventions. Recent advances in predictive analytics and machine learning have opened new avenues for improving recreational therapy delivery, with models effectively predicting participation in recreational and rehabilitation settings by analyzing demographics, psychological variables, and medical indicators to identify individuals at risk for disengagement (17). Among the algorithms applied, Random Forests, Gradient Boosting, and Naive Bayes have demonstrated high predictive accuracy in forecasting rehabilitation outcomes, with demonstrated potential for enabling customized, data-driven interventions tailored to residents’ cognitive and physical capabilities (18–20). However, significant gaps remain in the application of predictive modeling to veteran recreational therapy. No studies have developed comprehensive machine learning approaches specifically designed to predict RT engagement among veteran populations in long-term care settings. Furthermore, existing research has not adequately addressed methodological challenges such as class imbalance in participation prediction, where varying levels of engagement create complex classification problems that limit clinical utility of predictive models.

### 1.2 Objectives and contribution

This study addresses this critical gap by developing the first proof-of-concept machine learning approach to predict recreational therapy participation among veterans in long-term care facilities, with findings intended to establish methodological feasibility and inform subsequent multi-site validation rather than to provide immediately generalizable clinical tools. The research aims to: (1) develop robust predictive models for RT participation using algorithms optimized for healthcare datasets; (2) identify the most important factors influencing veteran participation patterns; (3) generate clinically interpretable decision rules for practical implementation; (4) address methodological challenges including class imbalance in participation prediction; and (5) provide evidence-based tools for optimizing intervention targeting and resource allocation in veteran recreational therapy programs. Through achieving these objectives, this research will provide clinical staff with validated, implementable tools for early identification of at-risk veterans while advancing the broader application of predictive modeling in long-term care settings.

## 2 Methods

### 2.1 Study design and setting

This study utilized a cross-sectional observational design incorporating machine learning methodologies to develop predictive models for recreational therapy (RT) engagement among veterans residing in long-term care facilities. The research was conducted at the New York State Veterans Home at Oxford (NYSVETS Home @ Oxford), a specialized state-operated facility that provides skilled nursing and therapeutic services to veteran populations. The setting offers a representative environment for examining participation patterns in institutional care contexts. All study procedures were reviewed and approved by the Binghamton University Institutional Review Board (IRB), and the study adhered to ethical standards for research involving older adults and other vulnerable populations, ensuring voluntary participation, informed consent, and data confidentiality.

### 2.2 Participants and recruitment

The target population comprised all veterans residing at NYSVETS Home @ Oxford who were eligible to participate in recreational therapy activities. Inclusion criteria specified residents aged 18 years or older with the cognitive capacity to provide informed consent and complete survey instruments. Cognitive function was assessed using the Brief Interview for Mental Status (BIMS), a validated tool administered by trained facility staff as part of standard care. The BIMS evaluates immediate recall, temporal orientation, and short-term memory, with scores ranging from 0 to 15. In accordance with CMS guidance, residents with a score of 7 or below — indicating severe cognitive impairment — were excluded to ensure informed consent capacity and reliable survey completion. Veterans with severe cognitive impairment precluding meaningful survey participation were excluded from the study. The NYSVETS Home has approximately 120 veteran residents at any given time. Of these, 76 residents met our inclusion criteria (aged 18+, cognitive capacity for informed consent). All eligible residents were invited to participate, representing a census sampling approach rather than probability sampling.

### 2.3 Data collection procedure

Data collection utilized a comprehensive self-administered survey instrument developed specifically for this investigation, titled the “Veterans Recreational Therapy Participation Survey.” The survey was administered electronically via Qualtrics, a Health Insurance Portability and Accountability Act (HIPAA)-compliant survey platform approved by Binghamton University. Participants were provided with detailed informed consent documentation prior to survey initiation, with explicit emphasis on voluntary participation and the right to withdraw without consequence to their care or services. The survey was designed to be completed in a single session lasting approximately 10–15 min to minimize participant burden while ensuring comprehensive data capture. Research staff provided assistance as needed while maintaining participant privacy and autonomy in response selection.

### 2.4 Survey instrument and variable measurement

The survey instrument was designed to capture comprehensive data on demographic characteristics, participation patterns, and activity preferences relevant to recreational therapy engagement predictive modeling. Demographic variables included age (categorized into eight groups from Under 30 to 91 + years), gender (Male, Female, Other, Prefer not to say), and length of residency at the facility (four categories ranging from <6 months to >2 years). Participation variables assessed self-reported frequency of RT program participation using four levels (Never, Low, Moderate, High) and duration of participation. Overall satisfaction with RT programs was measured using a 5-point Likert scale ranging from “Very dissatisfied” to “Very satisfied.” Activity preferences were captured through multiple-response formats assessing participation modalities (individual vs. group, indoor vs. outdoor preferences) and activity type preferences across eight categories: music therapy, physical exercise, group activities, art therapy, gardening, mind exercises, spiritual activities, and digital activities.

### 2.5 Variable operationalization for predictive modeling

#### 2.5.1 Dependent variables

Two binary outcome variables were constructed for predictive modeling based on self-reported participation frequency assessed via the question: “How frequently do you participate in RT programs at NYSVETS @ Oxford?” Response options were defined as: Never (no participation in past 3 months), Low (1–2 times per month or less), Moderate (1–2 times per week), and High (3 + times per week). Participants considered overall participation across all RT programs.

1. *High Participation* (binary): Participants reporting “High (regularly participate, 3 + times per week)” were coded as 1, while all other response categories (Never, Low, Moderate) were coded as 0. The clinical justification for this grouping reflects a fundamental distinction in recreational therapy practice between sustained, high-frequency engagement and other participation states. From an intervention standpoint, veterans achieving high participation represent the therapeutic ideal, defined as regular attendance at a frequency associated with meaningful physical, cognitive, and psychosocial benefits in long-term care settings. Veterans who participate never, infrequently, or only moderately share a common clinical need because they have not yet achieved or sustained the level of engagement associated with optimal therapeutic outcomes. The practical implication of this outcome is the identification of veterans for maintenance and optimization strategies. This enables recreational therapy staff to understand the factors that sustain high engagement and replicate those conditions proactively for other residents. Distinguishing between a veteran who attends once a month and one who attends twice a week is therefore of secondary clinical importance compared with identifying characteristics that predict consistent, high-frequency engagement. This grouping also addressed moderate class imbalance, with approximately 25 percent of participants classified as high participators and 75 percent classified as others. This distribution is consistent with the proportion of high-frequency recreational therapy engagement reported in comparable long-term care populations.
2. *Any Participation* (binary): Participants reporting any level of engagement, including Low (1 to 2 times per month), Moderate (1 to 2 times per week), or High (3 + times per week), were coded as 1, while those reporting “Never” were coded as 0. The clinical rationale for this grouping reflects a different and more urgent intervention objective focused on preventing complete disengagement. In recreational therapy practice, complete non-participation represents a critical clinical risk because veterans who disengage entirely lose access to the physical, psychological, and social benefits associated with recreational therapy programming. They also tend to become more difficult to re-engage once non-participation patterns become established. Veterans who maintain any level of participation, even if infrequent, retain a therapeutic connection to recreational therapy programming and remain accessible to engagement-enhancing interventions. In contrast, veterans who never participate often require more intensive outreach strategies, including barrier assessment, individualized engagement, and tailored activity matching. The practical implication of this outcome is the early identification of veterans who may require targeted intervention to prevent persistent disengagement. This grouping also addresses the severe class imbalance inherent in this outcome, where approximately 12.5 percent of residents reported no participation and 87.5 percent reported some level of participation. This pattern reflects the reality that complete non-participation, although clinically important, is relatively uncommon in this setting.

#### 2.5.2 Independent variables

*Demographic Predictors* were derived from survey responses and recoded for analytical purposes:

- Age groups were collapsed from eight original survey categories into four broader categories (Under 70, 71–80, 81–90, 91+) to ensure adequate sample size per group and reduce feature sparsity. Gender was binary-encoded with males coded as 1 and females/others as 0. Residency length was ordinally encoded from four original categories into three levels representing adaptation phases: New (<1 year), Established (1–2 years), and Long-term (>2 years). Complete demographic variable transformations are detailed in Supplementary Table S1.

*Activity Preference Predictors* were derived from multiple-response items:

- Eight activity categories (Music Therapy, Physical Exercise, Group Activities, Art Therapy, Gardening, Mind Exercises, Spiritual Activities, Digital Activities). These activity categories were identified through a review of existing recreational therapy programs at the study facility and other VA-affiliated LTC facilities, combined with evidence from prior literature on therapeutic engagement in older adults. Input from recreational therapy staff ensured that categories reflected the preferences and therapeutic needs of veterans. These categories encompass physical, social, creative, and spiritual/cognitive domains, providing comprehensive coverage of common RT modalities. An open-ended “other” option allowed participants to report preferences not captured by the predefined categories. These binary indicators formed the foundation for composite preference measures. Individual activity encoding specifications and selection frequencies are presented in Supplementary Table S2.
- Participation mode preferences were aggregated into four binary variables (Individual Outdoor, Individual Indoor, Group Outdoor, Group Indoor).

*Composite Feature Engineering* involved creating derived variables:

Total Preferences was calculated as the sum of all eight activity selections, while Preference Diversity represented the count of distinct activity categories selected. Due to the binary encoding approach, these measures yielded identical distributions in this dataset. Activity domain composites (Physical, Social, Creative, Spiritual/Mental) were created using maximum functions to capture broader therapeutic participation patterns, allowing veterans to be classified based on engagement with different types of therapeutic activities rather than specific individual preferences. Satisfaction scores were transformed from five-level Likert responses to numeric values, with missing data handled through mean imputation to maintain the complete dataset for modeling. Detailed composite feature calculations and statistical properties are provided in Supplementary Table S3, while satisfaction score transformation methodology is documented in Supplementary Table S4.

### 2.6 Machine learning methodology

#### 2.6.1 Analytical framework

**Figure 1:**
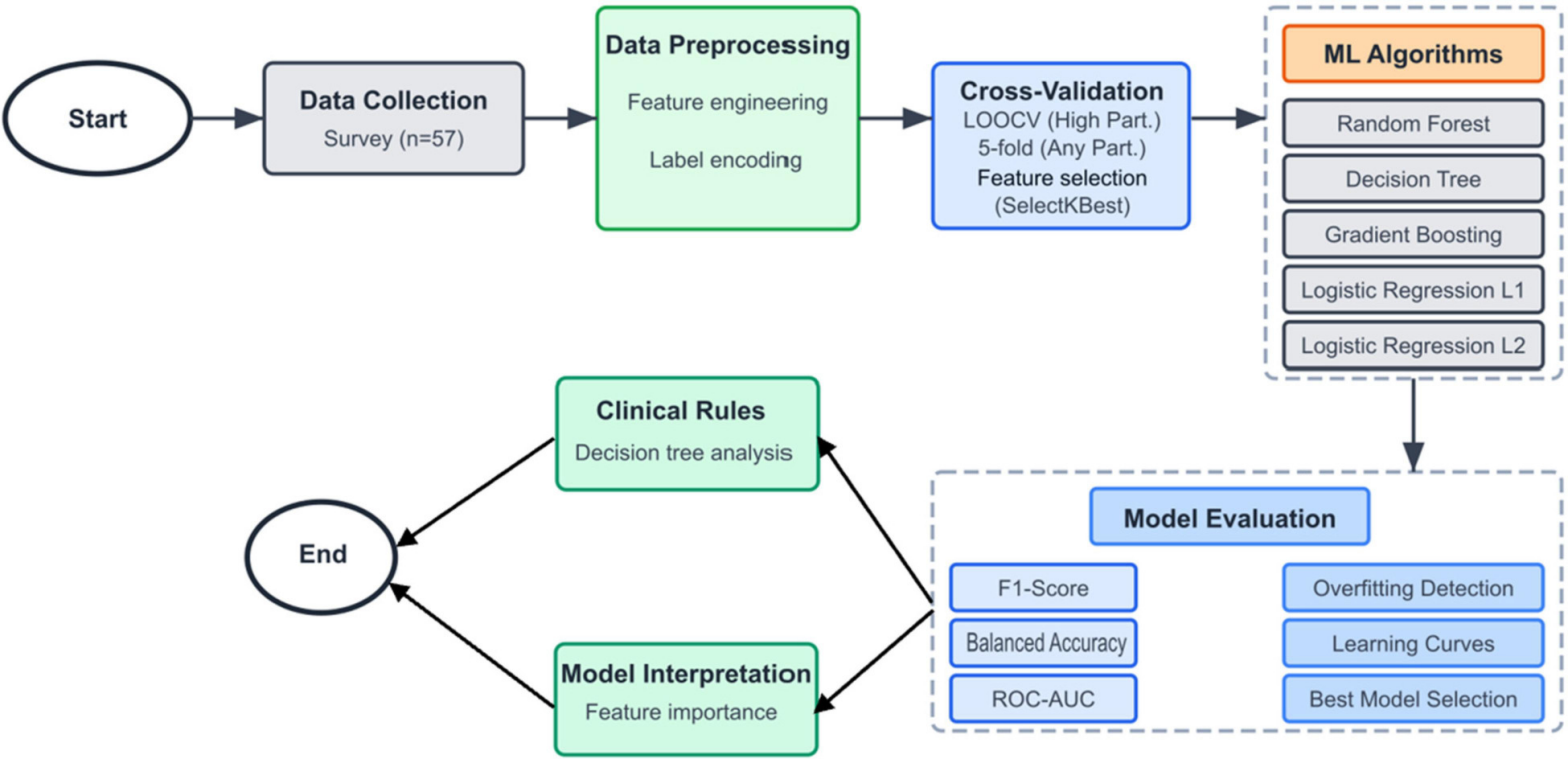
Machine learning methodology flowchart for recreational therapy participation prediction. The analytical framework incorporated systematic data preprocessing, outcome-specific cross-validation strategies, comprehensive algorithm evaluation, and clinical interpretation methods designed for small-sample healthcare applications.

Given the modest sample size (*n* = 57) relative to the feature space, a conservative machine learning approach was implemented to optimize model generalizability while preventing overfitting. The analytical framework prioritized model interpretability and clinical applicability over complex algorithmic sophistication. The complete machine learning methodology is summarized in Figure 1, which illustrates the systematic approach from data collection through model interpretation and clinical rule generation.

#### 2.6.2 Feature selection strategy

Statistical feature selection was implemented using a univariate analysis method that ranks predictors by their discriminative power for the outcome, measured via ANOVA F-statistics. Given the modest sample size (*n* = 57), the number of predictors was limited to a maximum of 6 in the final models to maintain an appropriate sample-to-variable ratio and reduce overfitting risk, consistent with recommendations for small-sample machine learning applications (21, 22). While the initial design allowed for up to 8 predictors, model performance optimization indicated that 6 predictors provided the best balance between accuracy and stability in this dataset. To ensure robust predictor identification and avoid data leakage, additional feature selection methods were implemented within each cross-validation fold, including mutual information selection, tree-based feature importance measures from Random Forest and Decision Tree models, and feature stability analysis across folds. Consensus features were determined based on stability metrics, reflecting consistent selection across methods and folds (Supplementary Table S6).

#### 2.6.3 Cross-validation methodology

*For High Participation Prediction*: Leave-One-Out Cross-Validation (LOOCV) was employed as the primary validation strategy to maximize training data utilization while providing unbiased performance estimates. LOOCV is particularly suitable for small datasets as it provides n-fold validation where each observation serves as a test case exactly once, yielding more stable performance estimates than traditional k-fold approaches with limited data (23, 24).

*Small-Sample Considerations for Performance Variability*: When LOOCV is applied to small datasets, each validation fold contains a single observation in the test set. Consequently, the prediction outcome for that individual observation can substantially influence the aggregated performance metric computed across folds. This characteristic often produces relatively large fold-to-fold variability in performance estimates, particularly for classification metrics derived from confusion-matrix counts such as the F1-score (25). Therefore, the standard deviation reported across folds should not be interpreted as direct evidence of model instability but rather as a reflection of sampling variability inherent to cross-validation in small datasets (25, 26). The precision of the mean performance estimate can instead be summarized using the standard error of the mean (SE = SD/√n), from which confidence intervals can be derived (27). To further quantify model stability, a permutation test (1,000 label permutations) was conducted to confirm that the observed performance exceeded chance levels (*p* < 0.05) (28), and bootstrapped confidence intervals were computed using 500 stratified resamples as a supplementary validation metric (29).

*For Any Participation Prediction*: Given the severe class imbalance (14% non-participants vs. 86% participants), 5-fold Stratified Cross-Validation was implemented to ensure proportional representation of both classes in each fold. Stratified sampling maintains the original class distribution across validation folds, preventing the creation of folds with only majority class samples that could lead to biased performance estimates (30). This approach provides more reliable evaluation of model performance on both participation classes while accommodating the limited sample size constraints.

#### 2.6.4 Class imbalance handling

The severe class imbalance (8 non-participants vs. 49 participants) for Any Participation Prediction, necessitated specialized handling to prevent models from defaulting to majority class prediction. Class weighting was implemented using scikit-learn's ‘balanced’ parameter, which automatically calculates inverse frequency weights to penalize minority class misclassification more heavily (31). This approach forces models to learn distinguishing patterns for both classes rather than achieving high accuracy through majority class prediction alone (30). For Gradient Boosting algorithms that lack native class weighting, sample weights were applied during training to achieve equivalent class balance correction (32).

#### 2.6.5 Algorithm selection and hyperparameter optimization

Five machine learning algorithms were systematically evaluated for both prediction tasks, with implementation approaches tailored to the specific characteristics of each outcome variable.

For High Participation prediction, standard algorithm implementations were employed given the observed class distribution of approximately 25% high participation and 75% non-high participation. Logistic Regression was implemented with both L1 (*α*=0.1) and L2 (*α*=1.0) regularization techniques (33, 34) to provide linear baseline models with different regularization approaches. For penalized logistic regression, we fixed the regularization parameter *α* to 0.1 for L1 and 1.0 for L2 regularization rather than conducting extensive hyperparameter tuning. This choice follows standard methodological recommendations in the penalized regression literature (33, 34) which suggest adopting conservative defaults in small-sample healthcare applications to minimize overfitting risk and maintain clinically interpretable models. Aggressive hyperparameter tuning using leave-one-out cross-validation was avoided because, in modest sample sizes, it may yield unstable estimates and overfit to noise rather than capturing generalizable patterns. Random Forest was configured with 50 estimators, maximum depth of 3, minimum samples per leaf of 3, and minimum samples for splitting of 5 to prevent overfitting while maintaining predictive capability. Decision Tree implementation employed restrictive pruning parameters with maximum depth of 3, minimum samples per leaf of 5, and minimum samples for splitting of 10 to ensure model simplicity and interpretability. Gradient Boosting was applied with modest complexity settings including 50 estimators, learning rate of 0.1, and maximum depth of 2 to balance predictive capability with generalization.

For Any Participation prediction, class-weighted implementations were employed to address the severe class imbalance. Class-weighted Logistic Regression was implemented with both L1 and L2 regularization using the ‘balanced’ class weight parameter to automatically penalize minority class misclassification. Class-weighted Random Forest utilized balanced class weights combined with the same conservative hyperparameters as the standard implementation to maintain model stability while addressing class imbalance. Class-weighted Decision Tree employed balanced class weights with restrictive pruning parameters to ensure both class balance correction and model interpretability. Gradient Boosting was implemented with sample weighting equivalent to balanced class weights applied during training to achieve similar class balance correction.

All hyperparameters were deliberately set to conservative values across both prediction tasks to reduce the risk of overfitting given the limited sample size of 57 participants, prioritizing model stability and generalizability over complex algorithmic optimization. This strategy reflects an explicit bias–variance trade-off: constraining model complexity reduces variance in cross-validation estimates but may introduce some degree of bias, meaning that certain subtle predictive patterns in the data may remain unmodeled. Consequently, the reported performance metrics should be interpreted as conservative estimates of model capability under the chosen parameter constraints. The implications of this trade-off are discussed further in Section 4.3.

#### 2.6.6 Model evaluation and performance assessment

*Primary Performance Metrics*:

- For High Participation: F1-score was designated as the primary evaluation metric due to its balanced consideration of precision and recall, particularly important given the moderate class imbalance (35).
- For Any Participation: Balanced accuracy was used as the primary metric due to its equal weighting of sensitivity and specificity, providing more meaningful assessment when class distributions are severely skewed (14% vs. 86%) (36).

*Overfitting Detection*: Learning curves were systematically generated for all algorithms to assess generalization capacity using the primary evaluation metric (F1-score for High Participation, balanced accuracy for Any Participation). Training-validation performance gaps exceeding 15% were considered indicative of overfitting, with models exceeding this threshold labeled as “Overfitting” in performance summaries.

*Statistical Inference*: All performance estimates were accompanied by standard deviations derived from cross-validation distributions. Model selection was based on mean primary metric performance across all cross-validation folds, with consideration of both performance magnitude and stability (lower standard deviation indicating more consistent performance).

*Supplementary Holdout Evaluation*: In addition to the primary Leave-One-Out Cross-Validation (LOOCV) analysis, a supplementary stratified random 70/30 split of the dataset was conducted as a *post hoc* descriptive analysis to illustrate model behavior under a conventional train–test scenario. In this split, 39 observations were used for model training and 18 observations were reserved for testing while preserving the observed class distribution. Because the holdout samples were drawn from the same dataset used for the LOOCV procedure, this evaluation is descriptive rather than a strictly independent validation. The primary performance estimate reported in this study remains the LOOCV F1-score.

#### 2.6.7 Feature importance and model interpretability

To enhance clinical applicability and provide actionable insights for recreational therapy program development, feature importance analysis was conducted on the best-performing models for each prediction task. Gini impurity-based importance scores were extracted using the feature_importances_ attribute, which measures the total decrease in node impurity weighted by the probability of reaching each node across all trees in the ensemble (37). Feature importance scores were automatically normalized by the Random Forest algorithm and ranked in descending order to identify the most influential predictors (38). The top-ranked features from each model were interpreted in the context of clinical practice to provide actionable insights for recreational therapy program design and targeted intervention strategies. This approach ensures that predictive models serve not only as classification tools but also as interpretable frameworks for understanding the underlying mechanisms driving veteran participation in recreational therapy activities. The analysis focused on identifying the relative contribution of demographic characteristics, facility factors, and activity preferences to participation prediction, enabling evidence-based program development and resource allocation decisions.

To complement the feature importance analysis, simplified Decision Tree models were constructed specifically for generating explicit “if-then” clinical rules (39). These interpretable models were configured with restrictive hyperparameters to ensure clinical utility: maximum depth of 4 levels, minimum samples per split of 5, and minimum samples per leaf of 3. A streamlined feature set focusing on readily assessable clinical variables (age, gender, residency length, activity preferences, satisfaction scores) was employed to ensure practical applicability. Decision pathways were systematically extracted from the trained trees and translated into clinical language, with participation probability estimates calculated for each terminal node. The generated rules provide explicit decision criteria that recreational therapy staff can apply during routine assessments to identify veterans most likely to benefit from specific intervention strategies. All terminal node probability estimates derived from the decision tree analysis are reported alongside exact binomial (Clopper-Pearson) 95% confidence intervals throughout the work to appropriately characterize statistical uncertainty arising from small subgroup sizes (40).

#### 2.6.8 Software and implementation environment

All analyses were conducted in Python (version 3.10) using packages including scikit-learn, pandas, NumPy, and matplotlib. Feature selection was performed using the SelectKBest function with f_classif scoring from scikit-learn (41). The analyses were implemented in a secure computing environment to ensure data integrity and reproducibility.

## 3 Results

### 3.1 Participant characteristics and data quality

Of the 76 individuals invited, 57 (75%) consented and completed the survey. The sample was predominantly older male veterans, with 82.1% aged 71 or older and 78.6% male. The average length of stay was 19.3 months, with over half residing in the facility for more than one year. Most respondents reported being satisfied or very satisfied with recreational therapy programs. Full demographic details are presented in Table 1.

**Table 1:** Summary of participant characteristics (*N* = 56).

Variable	Category	Percentage (%)	95% Confidence interval
Age group^a^	31–40 years	5.4	1.1–14.9
	41–50 years	1.8	0.0–9.6
	51–60 years	1.8	0.0–9.6
	61–70 years	8.9	3.0–19.6
	71–80 years	41.1	28.1–55.0
	81–90 years	26.8	15.8–40.3
	91 + years	14.3	6.4–26.2
Gender^a^	Male	78.6	66.5–87.8
	Female	19.6	10.2–32.4
	Prefer not to say	1.8	0.0–9.6
Length of stay^a^	<6 months	10.7	4.0–21.9
	6 months – 1 year	28.6	17.3–42.2
	1–2 years	30.4	18.8–44.1
	>2 years	30.4	18.8–44.1
Satisfaction with RT programs^b^	Very satisfied	22.0	11.5–36.0
	Satisfied	38.0	24.7–52.8
	Neutral	26.0	14.6–40.3
	Dissatisfied	14.0	5.8–26.7

a One participant did not respond to demographic questions, resulting in *n* = 56 for all demographic variables.

b Among participants only, *n* = 50.

### 3.2 Outcome variable distribution

Two primary outcome variables were created for modeling purposes, derived from self-reported participation frequency in recreational therapy programs. High participation, defined as regularly engaging in activities, was reported by 14 participants (25.0%). In contrast, participation, including low, moderate, and high levels—was reported by 49 participants (87.5%), while only 7 participants (12.5%) reported never engaging in recreational therapy. This distribution highlights a class imbalance, commonly observed in healthcare-related prediction tasks, and especially relevant when modeling high participation behavior as a distinct outcome. Detailed frequencies and confidence intervals for all response categories are presented in Table 2.

**Table 2:** Self-reported frequency of participation in recreational therapy activities (*N* = 56).

Characteristic	Response option	*n*	%	95% CI
Frequency of participation^a^	Never	7	12.5	5.1–24.1
	Low (rarely participate)	21	37.5	25.0–51.2
	Moderate (occasionally participate)	14	25.0	14.4–38.4
	High (regularly participate)	14	25.0	14.4–38.4

a One participant did not respond to this question, resulting in *N* = 56.

### 3.3 Feature engineering and model development

A comprehensive feature engineering approach yielded 18 initial predictor variables encompassing demographic characteristics, activity preferences, and composite measures. Demographic features included age (numeric encoding), gender (binary), and residency length (ordinal encoding). Eight binary indicators captured specific activity preferences including music therapy, physical exercise, group activities, art therapy, gardening, mind exercises, spiritual activities, and digital activities. Composite features comprised total preference count, preference diversity, and grouped activity categories representing physical, social, creative, and spiritual/mental domains.

Statistical feature selection using SelectKBest with f_classif scoring was implemented to address the modest sample size relative to the feature space. Feature selection was constrained to a maximum of six variables per model to maintain appropriate sample-to-predictor ratios, adhering to established guidelines for small-sample machine learning applications. The selected features and their respective F-scores for both the high participation and any participation models are presented in Table 3.

**Table 3:** Top predictive features identified using SelectKBest with *f_classif* scoring.

Outcome variable	Selected feature	F-score
High participation	Preference_Diversity	18.94
	Total_Preferences	18.94
	Group_Activities_Selected	16.58
	Spiritual_Activities_Selected	8.68
	Residency_Numeric	8.09
	Social_Preference	7.19
Any participation	Residency_Numeric	2.35
	Digital_Activities_Selected	1.29
	Preference_Diversity	1.20
	Total_Preferences	1.20
	Gender_Male	1.12
	Spiritual_Activities_Selected	1.10

### 3.4 Model performance and validation

Five machine learning algorithms were systematically evaluated using LOOCV to maximize training data utilization while providing unbiased performance estimates. Algorithms included logistic regression with L1 and L2 regularization, Random Forest, Decision Tree, and gradient boosting, all configured with conservative hyperparameters to prevent overfitting in the small-sample context.

#### 3.4.1 High participation prediction

For high participation prediction, Random Forest emerged as the optimal algorithm, achieving a mean LOOCV F1-score of 0.860 ± 0.347. While Random Forest and Decision Tree achieved identical cross-validation performance, Random Forest was selected for its superior generalization properties and more reliable feature importance estimates typical of ensemble methods. The model demonstrated robust performance with an accuracy of 0.860 ± 0.347 and maintained good generalization with an overfitting gap of only 0.030, well below the 0.15 threshold for concerning overfitting (Table 4). The coincidence of accuracy and F1-scores reflects the specific prediction patterns across LOOCV folds, where precision and recall were identical in this dataset, and was verified through independent recalculation.

**Table 4:** Machine learning model performance for high participation prediction (*n* = 57).

Algorithm	LOOCV accuracy (±SD)	LOOCV F1-score (±SD)	Overfitting gap	Model status
Random Forest	0.860 ± 0.347	0.860 ± 0.347	0.030	Best model
Decision Tree	0.860 ± 0.347	0.860 ± 0.347	0.030	Good
Gradient Boosting	0.807 ± 0.395	0.807 ± 0.395	0.173	Overfitting
Logistic Regression (L2)	0.789 ± 0.408	0.789 ± 0.408	0.109	Good
Logistic Regression (L1)	0.702 ± 0.457	0.702 ± 0.457	0.005	Good

In a supplementary stratified holdout split (*n* = 18), the final Random Forest model achieved an accuracy of 83.3%. Because this split was derived from the same dataset used in the LOOCV procedure, the result is reported as a descriptive comparison rather than independent validation.

##### 3.4.1.1 Model validation and overfitting assessment

**Figure 2:**
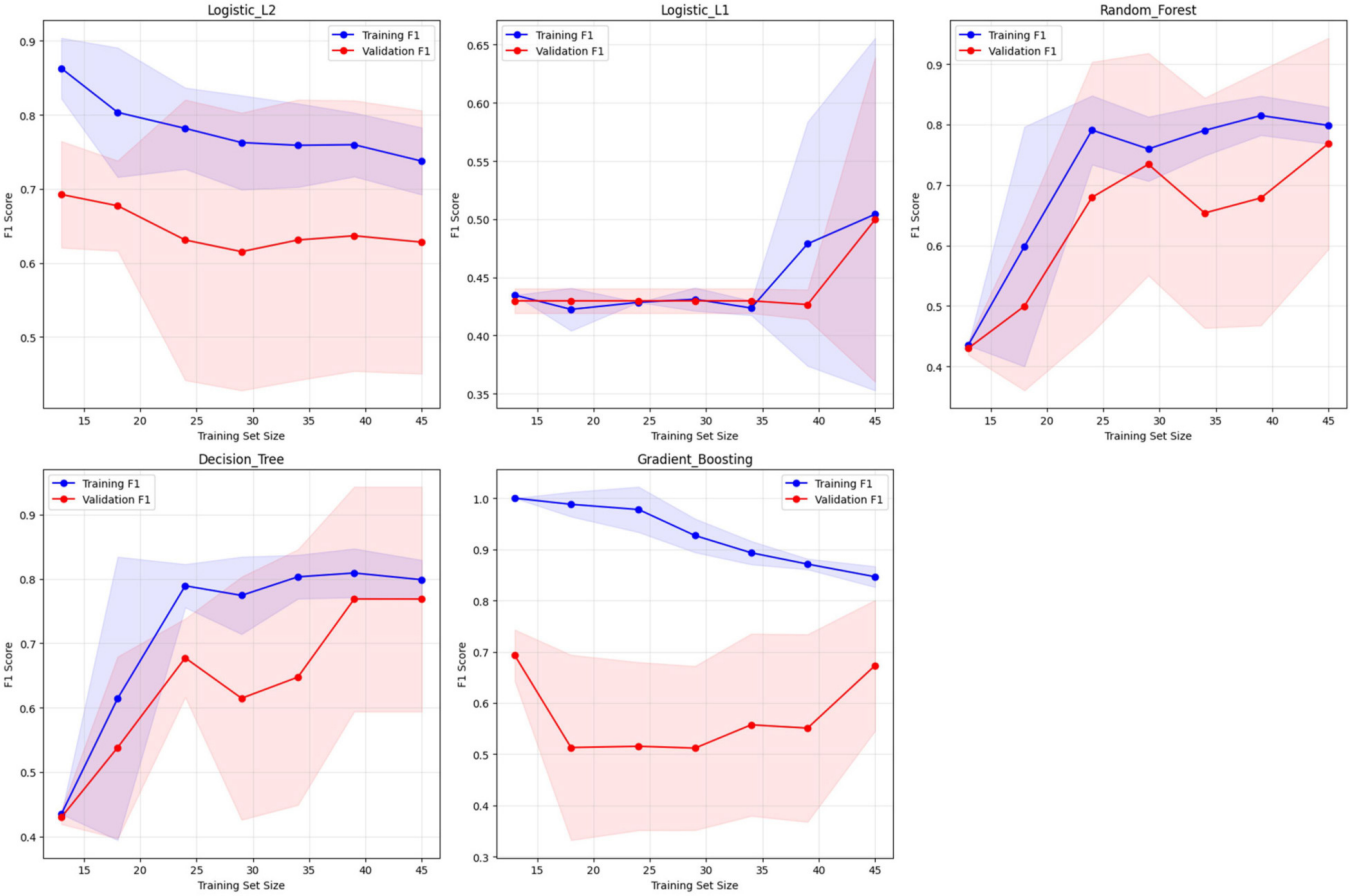
Learning curves for overfitting detection - high participation prediction [learning curves showing training vs. validation F1-scores using adaptive cross-validation (5-fold when possible, adjusted for smaller sample sizes) across training set sizes from 30% to 100% of available data (*n* = 57)].

Learning curve analysis confirmed appropriate model complexity for the dataset size across all algorithms (Figure 2). The curves demonstrate convergence between training and validation performance, with most algorithms showing stable generalization. Gradient Boosting exhibited the largest training-validation gap (0.173), indicating potential overfitting, while Random Forest and Decision Tree maintained excellent stability with gaps of only 0.030. The learning curve analysis also revealed evidence of underfitting in the Logistic Regression (L1) model, characterized by consistently low performance across all training set sizes and a minimal training-validation gap (0.005). This pattern suggests that L1 regularization may be overly restrictive for this dataset, where the aggressive feature selection inherent in L1 penalties appears to sacrifice predictive capability by eliminating potentially informative variables.

##### 3.4.1.2 Comprehensive model performance analysis

**Figure 3:**
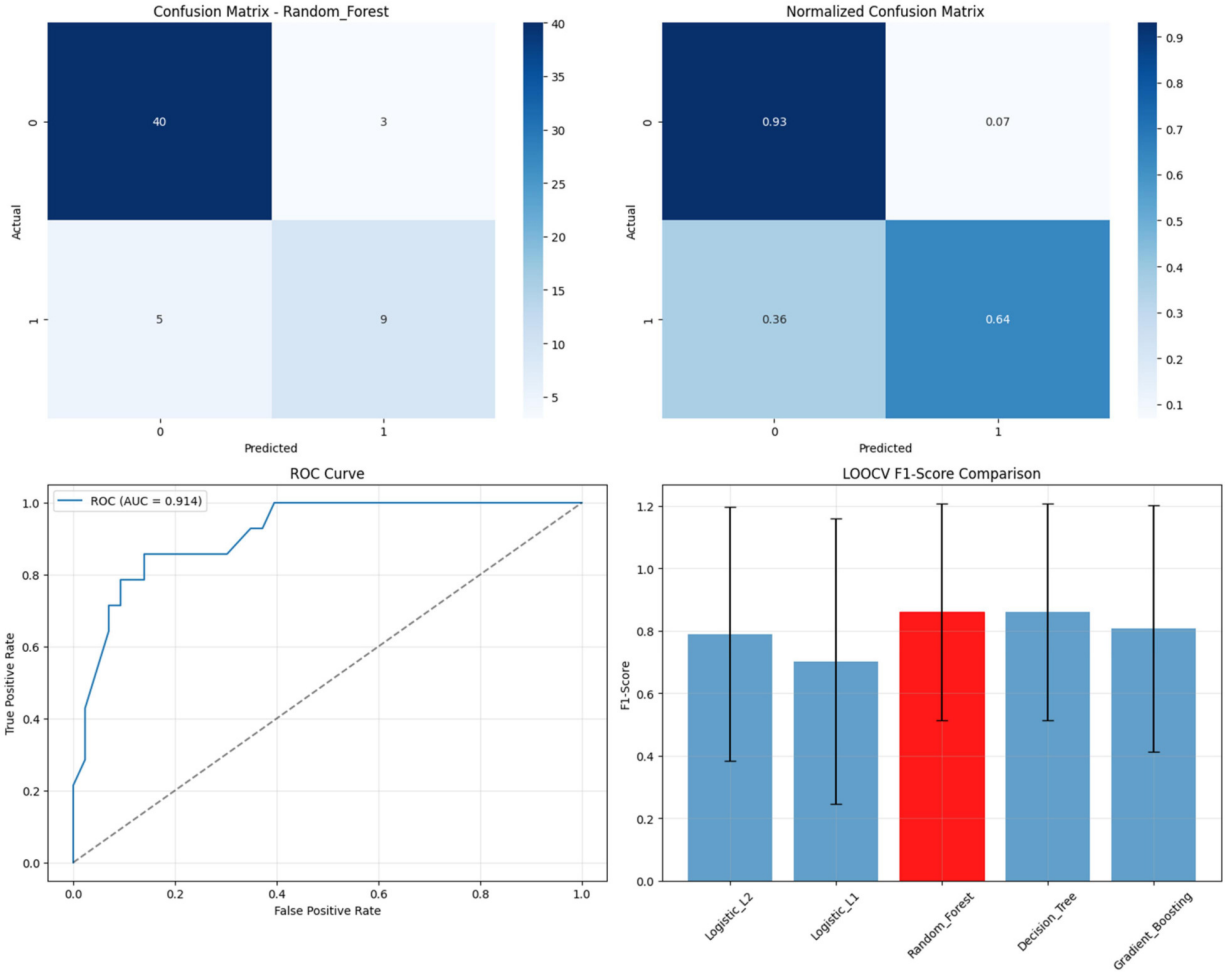
Random forest model performance - high participation prediction (four-panel figure showing the raw confusion matrix, normalized confusion matrix, ROC curve with AUC = 0.914, and LOOCV F1-score comparison across all algorithms with Random Forest highlighted as best performer).

The Random Forest model demonstrated robust classification performance with strong discriminative ability (Figure 3). The confusion matrix revealed excellent specificity (93% correct identification of non-high participants) and moderate sensitivity (64% correct identification of high participants). The ROC curve analysis yielded an AUC of 0.914, indicating excellent discrimination between high and non-high participation cases.

##### 3.4.1.3 Model stability and statistical validation

To further assess the robustness of the Random Forest model under the small sample size constraint (*n* = 57), additional statistical validation analyses were conducted. The model achieved a mean LOOCV F1-score of 0.860 with a standard deviation of 0.347 across folds. Because LOOCV evaluates one observation per test fold, variability across folds can be amplified in small datasets. To quantify the precision of the mean estimate, the standard error of the mean was calculated (SE = SD/√*n* = 0.347/√57 = 0.046), yielding a 95% confidence interval of [0.770, 0.950] for the mean F1-score. To determine whether the observed predictive performance exceeded chance-level classification, a permutation test was conducted using 1,000 label permutations. The null distribution produced a mean F1-score of 0.381 with a maximum value of 0.783 across all permutations. The observed F1-score of 0.860 exceeded this maximum and corresponded to a permutation *p*-value of 0.018, indicating that the model performance is unlikely to arise from random label assignments.

An additional bootstrap resampling analysis (500 stratified iterations preserving class proportions) was performed to obtain an assumption-independent estimate of performance variability. The bootstrap procedure produced a mean F1-score of 0.853 and a 95% percentile confidence interval of [0.741, 0.947], which closely aligns with the LOOCV-derived interval. This consistency across validation approaches indicates stable model performance under repeated sampling of the dataset.

Taken together, the SE-based confidence interval, permutation significance test, and bootstrap validation results, along with the small overfitting gap observed in the learning curve analysis (0.030), provide convergent evidence supporting the stability and reliability of the Random Forest model's predictive performance despite the modest sample size. Full results of the permutation and bootstrap analyses are reported in Supplementary SF 2.

#### 3.4.2 Any participation prediction

For any participation prediction, given the severe class imbalance (14% non-participants vs. 86% participants), class-weighted machine learning algorithms were implemented to ensure meaningful prediction of both participation classes. Random Forest with balanced class weights achieved optimal performance with a mean balanced accuracy of 0.619 ± 0.081 using 5-fold stratified cross-validation. The model demonstrated excellent ability to identify both participants and non-participants, addressing the clinical need for targeted intervention identification (Table 5).

**Table 5:** Class-weighted machine learning model performance for any participation prediction (*n* = 57).

Algorithm	Balanced accuracy (±SD)	Sensitivity	Specificity	F1-score	Model status
Random forest weighted	0.619 ± 0.081	0.838	0.400	0.537	Best model
Decision tree weighted	0.588 ± 0.099	0.576	0.600	0.485	Good
Gradient boosting weighted	0.578 ± 0.087	0.756	0.400	0.505	Good
Logistic regression (L2) weighted	0.538 ± 0.162	0.676	0.400	0.456	Good
Logistic regression (L1) weighted	0.500 ± 0.000	0.000	1.000	0.121	Poor

Accuracy represents the proportion of correctly classified observations. Precision represents the proportion of predicted positive cases that were correctly identified. Sensitivity (recall) represents the model's ability to correctly identify participants. Specificity represents the model's ability to correctly identify non-participants. The F1-score represents the harmonic mean of precision and sensitivity, providing a balanced measure of model performance when class distributions are uneven.

##### 3.4.2.1 Model validation and overfitting assessment

**Figure 4:**
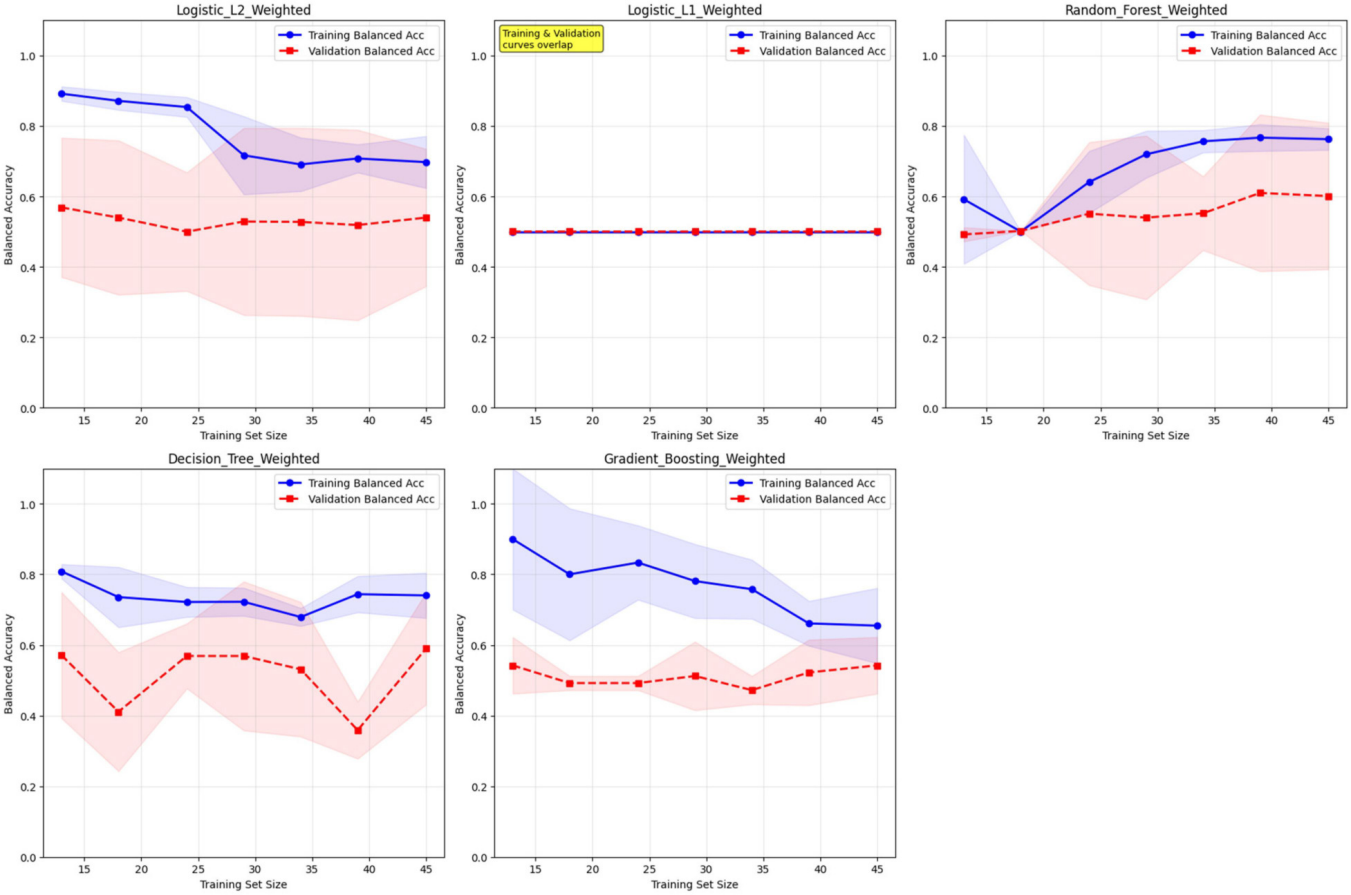
Learning curves for class-weighted models - any participation prediction learning curves showing training vs. validation balanced accuracy across different training set sizes for all five class-weighted algorithms, demonstrating model stability and appropriate handling of class imbalance.

The severe class imbalance (8 non-participants vs. 49 participants) necessitated specialized handling to prevent models from defaulting to majority class prediction. Class weights were automatically calculated using scikit-learn's ‘balanced’ parameter, which applies inverse frequency weighting (Class 0 weight: 3.56, Class 1 weight: 0.58). This approach penalizes misclassification of the minority class (non-participants) more heavily, forcing models to learn distinguishing patterns rather than defaulting to majority class prediction. Learning curve analysis confirmed stable model performance across different training set sizes (Figure 4). The Random Forest model showed excellent generalization with minimal overfitting, while maintaining the critical ability to identify non-participants who may benefit from targeted interventions to increase engagement.

##### 3.4.2.2 Comprehensive model performance analysis

**Figure 5:**
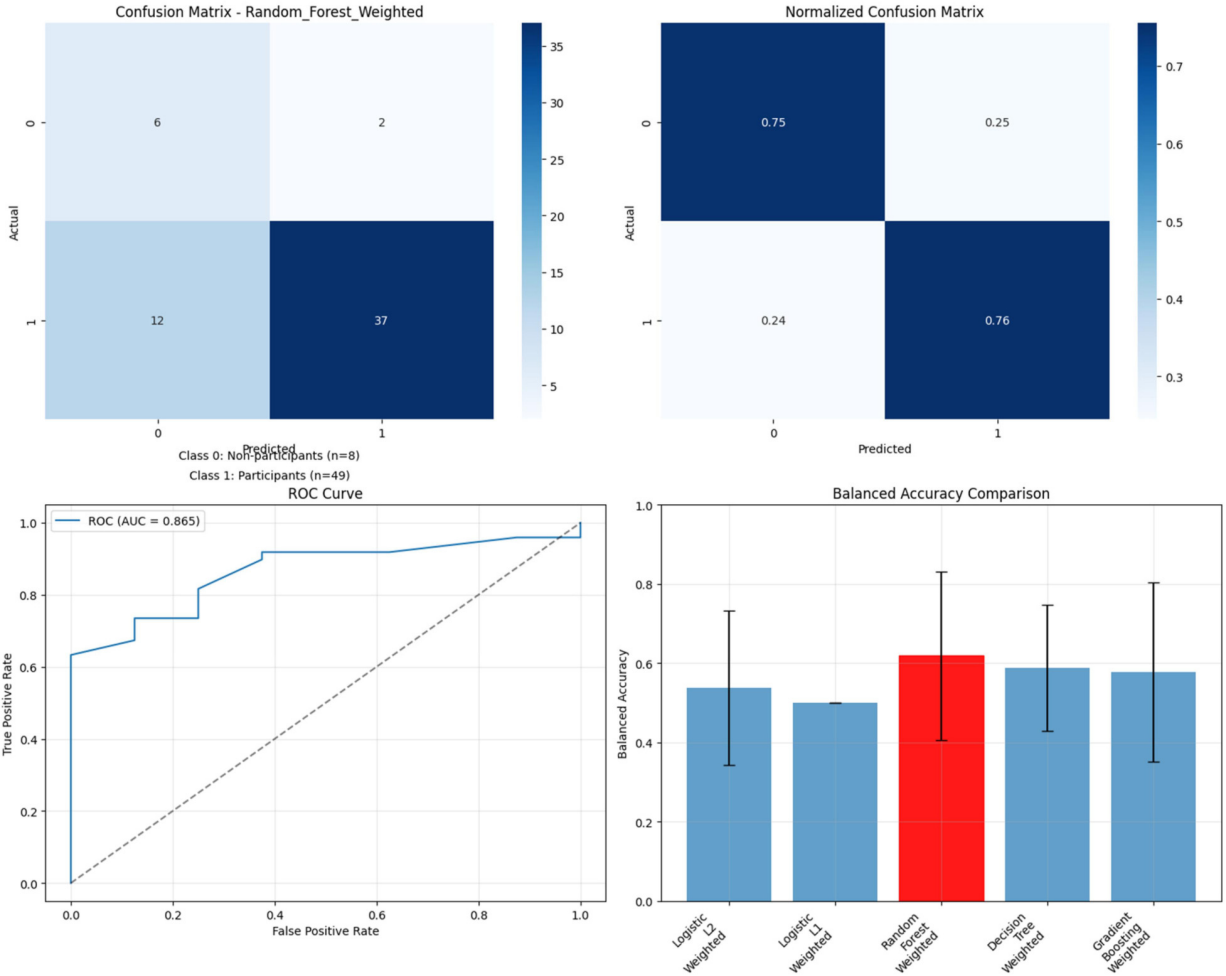
Class-weighted Random Forest model performance - any participation prediction. Four-panel figure showing the raw confusion matrix demonstrating successful identification of both classes, normalized confusion matrix showing balanced performance, ROC curve with AUC = 0.865, and balanced accuracy comparison across all class-weighted algorithms with Random Forest highlighted as best performer.

The class-weighted Random Forest model demonstrated clinically meaningful performance with balanced prediction capabilities (Figure 5). The confusion matrix revealed the model's ability to identify both classes: 75% of non-participants and 76% of participants were correctly classified. This represents a substantial improvement over unweighted models that achieved high overall accuracy by predicting all cases as participants but failed to identify any non-participants. The ROC curve analysis yielded an AUC of 0.865, indicating excellent discrimination between participants and non-participants. Critically, the model achieved 40% specificity (ability to identify non-participants) while maintaining 84% sensitivity (ability to identify participants), providing a clinically useful balance for intervention targeting.

### 3.5 Feature importance analysis

Feature importance analysis revealed distinct predictive patterns for each participation outcome, providing clinically actionable insights into the factors driving recreational therapy engagement among veterans.

#### 3.5.1 High participation prediction

**Figure 6:**
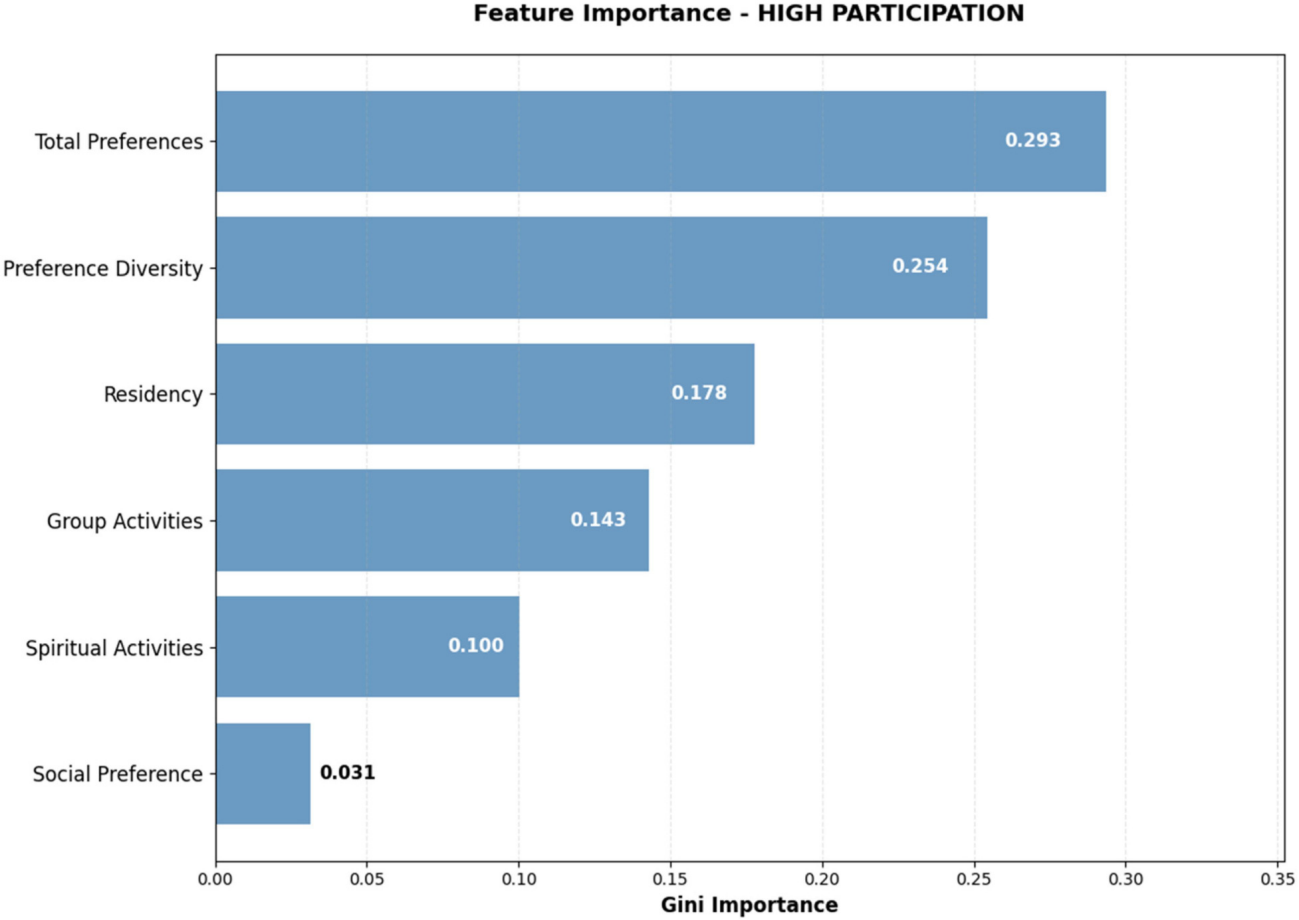
Feature importance analysis - high participation prediction Gini importance scores from Random Forest model showing relative contribution of each predictor variable to high participation classification. Values represent normalized importance scores summing to 1.0.

Random Forest feature importance analysis for high participation identified activity preference patterns as the primary drivers of sustained engagement (Figure 6). Total Preferences emerged as the most influential predictor (Gini importance: 0.293), indicating that veterans with broader activity interests demonstrate significantly higher likelihood of regular participation. Preference Diversity ranked second (0.254), reinforcing the importance of varied recreational interests in sustaining high-level engagement. Length of residency (0.178) ranked third in importance, suggesting that longer-term residents develop greater familiarity and comfort with available programming. Group Activities preference (0.143) and Spiritual Activities preference (0.100) demonstrated moderate importance, while Social Preference composite showed minimal influence (0.031) on high participation prediction.

#### 3.5.2 Any participation prediction

**Figure 7:**
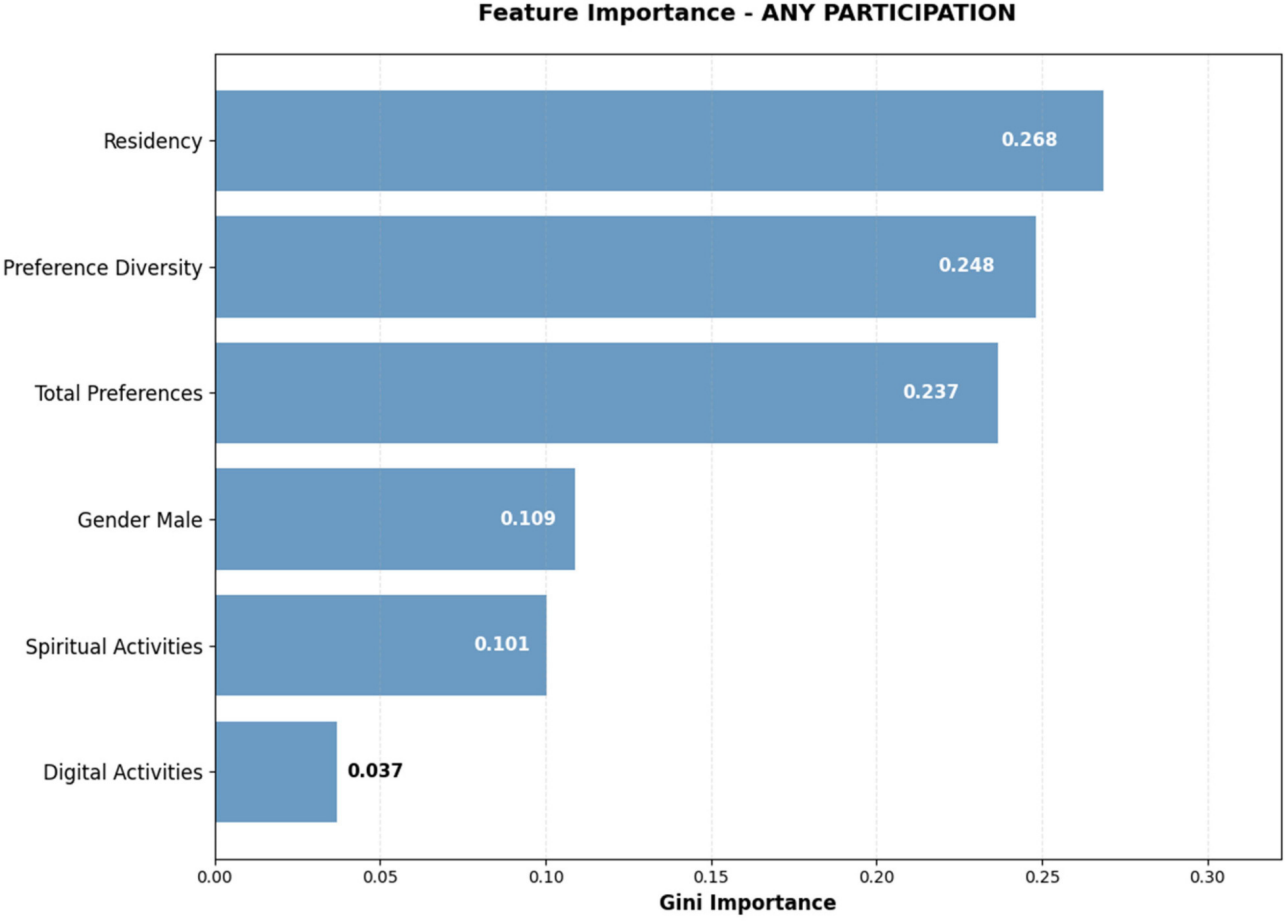
Feature importance analysis - any participation prediction Gini importance scores from class-weighted Random Forest model demonstrating the relative contribution of each predictor to any participation classification. The prominence of residency length highlights the importance of facility tenure in engagement patterns.

Class-weighted Random Forest analysis for any participation revealed a different pattern of influential factors (Figure 7). Residency length emerged as the primary predictor (Gini importance: 0.268), indicating that facility tenure is the strongest determinant of any level of recreational therapy engagement. This finding suggests that early intervention strategies may be particularly critical for new residents who have not yet established participation patterns. Preference Diversity (0.248) and Total Preferences (0.237) ranked as the second and third most important factors, demonstrating consistent influence of activity interest breadth across both participation outcomes. Gender (Male) showed moderate importance (0.109), while specific activity preferences including Spiritual Activities (0.101) and Digital Activities (0.037) contributed to prediction accuracy but with lower relative importance.

#### 3.5.3 Clinical decision rules from interpretable decision tree

For clinical rule generation, we employed a simplified feature set comprising readily assessable variables (age, gender, residency length, total preferences, satisfaction scores) rather than the statistically-selected features from our Random Forest models (feature set comparison detailed in Supplementary Table S5). This approach prioritized clinical implementation feasibility, as these variables are universally available during routine assessments without requiring comprehensive surveys. The complete decision tree diagram is provided in Supplementary Figure S1. A simplified Decision Tree analysis was conducted using clinically-accessible variables to generate explicit clinical rules for high participation prediction that can be directly applied in recreational therapy practice. The decision tree analysis revealed clear pathways to high recreational therapy participation based on readily assessable veteran characteristics.

##### 3.5.3.1 Primary decision pathway - high participation predictors

The decision tree identified two primary pathways to high recreational therapy participation:

1. *High Engagement Pathway*: Veterans with preference diversity >4.5 activities, satisfaction scores >3.84, and age ≤81 years demonstrated 100% observed probability of high participation [*n* = 5; 95% exact binomial CI: (47.8%, 100%)]. This represents veterans with broad recreational interests, high program satisfaction, and younger-old age profiles.
2. *Moderate Engagement Pathway*: Veterans with preference diversity >4.5 activities, satisfaction scores >3.84, but age >81 years showed 67% observed probability of high participation [*n* = 3; 95% exact binomial CI: (9.4%, 99.2%)], indicating that advanced age may moderate but not eliminate high engagement potential.

##### 3.5.3.2 Low participation risk factors

The tree identified several clear risk factors for low/no participation:

- *Limited Preference Diversity (≤4.5 activities)* combined with low satisfaction (≤4.5) resulted in consistently low participation probability across all residency and demographic subgroups.
- *New residents (≤1.5 years)* with limited preferences showed particularly low engagement [observed probability: 8.3%; *n* = 12; 95% exact binomial CI: (0.2%, 38.5%)].
- *Moderate satisfaction (3.5–4.5)* with limited preferences led to low participation regardless of other factors.

##### 3.5.3.3 Actionable clinical rules

Based on the decision tree analysis, the following clinical screening rules emerge:

1. *High Priority for Engagement*: Veterans with >4.5 activity preferences and satisfaction >3.84.
2. *Intervention Targets*: Veterans with ≤4.5 activity preferences and satisfaction ≤4.5, especially new residents.
3. *Age Considerations*: Veterans >81 years may benefit from modified approaches even with high preferences and satisfaction.

Feature importance analysis from the decision tree aligned with the Random Forest results, reinforcing the significance of key predictors: Preference Diversity emerged as the primary discriminator (importance = 0.655), followed by Satisfaction Score as a secondary factor (0.232), Residency Length as a contextual modifier (0.078), and Age as a minor influence (0.035).

## 4 Discussion

### 4.1 Principal findings

This study is the first to apply a comprehensive machine learning approach for predicting recreational therapy participation among veterans in long-term care facilities. The approach achieved clinically meaningful predictive accuracy and addressed critical methodological challenges in healthcare prediction modeling (42, 43). The Random Forest algorithm proved to be the most effective method for both prediction tasks, achieving a strong F1-score of 0.860 in predicting high participation, and balanced accuracy of 0.619 for any participation prediction—despite the presence of severe class imbalance in that task. These results are comparable to those reported in similar healthcare engagement prediction studies and reinforce the value of Random Forest for clinical classification tasks (44, 45). Balanced accuracy was used to ensure equitable evaluation across both classes, given the disproportionate number of participants vs. non-participants. The findings reveal distinct predictive patterns that offer important insights into the mechanisms driving recreational therapy engagement among veteran populations and can support early identification of at-risk individuals for targeted intervention.

In predicting high participation, the diversity and breadth of activity preferences were the most influential factors, with total preferences and preference diversity contributing to more than half of the model's overall predictive power. This finding aligns with theoretical frameworks of successful aging that emphasize the importance of diverse leisure engagement for maintaining physical, cognitive, and social functioning in older adults (46). The prominence of group activities and spiritual activities as significant predictors highlights the multidimensional benefits of recreational therapy (47–49). These activities support both social connection and meaning making, which are especially important for veteran populations navigating identity transitions in institutional care setting (50, 51). For any participation prediction, facility tenure emerged as the strongest predictor, accounting for 26.8% of the model's discriminative capability. This finding suggests that the initial period following admission represents a critical window for engagement intervention, consistent with adaptation theories that highlight the importance of early institutional adjustment for long-term outcomes (52). The sustained importance of preference diversity and total preferences across both prediction tasks reinforces the fundamental role of individual activity interests in driving participation, while the moderate influence of gender suggests that demographic factors play secondary but meaningful roles in engagement patterns.

### 4.2 Clinical implications and practical applications

The development of validated predictive models for recreational therapy engagement addresses a critical gap in evidence-based long-term care practice. The clinical decision rules derived from interpretable decision tree analysis provide immediately actionable screening criteria that recreational therapy staff can implement during routine assessments. Veterans demonstrating high preference diversity (>4.5 activities) combined with high satisfaction scores (>3.84) represent optimal candidates for sustained high-level engagement, while those with limited preferences (≤4.5) and moderate satisfaction (≤4.5) require targeted interventions to prevent disengagement.

The identification of facility tenure as the primary predictor of any participation has profound implications for intervention timing and resource allocation. New residents (≤1.5 years) with limited activity preferences represent the highest-risk population for non-participation, suggesting that early engagement protocols should be prioritized during the initial adaptation period. This finding supports the implementation of structured orientation programs that introduce new residents to a range of recreational options while assessing individual preferences and potential barriers to engagement. Such programs align with person-centered care models that emphasize individualized leisure experiences (6), foster meaningful self-directed activity (53), and can be enhanced through structured, technology-supported delivery (8). The prominence of preference diversity as a consistent predictor across both outcome measures suggests that recreational therapy programs should prioritize offering varied activity options rather than focusing intensively on single modalities (14). This has direct implications for program design, staffing allocation, and facility resource investment. Programs that can accommodate diverse interests through music therapy, physical exercise, group activities, art therapy, gardening, mind exercises, spiritual activities, and digital programming are more likely to achieve sustained engagement across heterogeneous veteran populations (47, 49).

### 4.3 Limitations and future directions

Several limitations should be acknowledged when interpreting these findings, presented here in order of their potential impact on the study's primary conclusions. The most consequential limitation is the modest sample size (*n* = 57), which directly constrains the statistical power and generalizability of the predictive models. While the conservative machine learning approach, LOOCV validation strategy, and supplementary stability analyses (permutation testing, bootstrap resampling) were specifically designed to address small-sample challenges, the limited observations prevent application of more sophisticated ensemble methods and reduce the precision of subgroup-level probability estimates derived from decision tree terminal nodes. Future studies with larger samples would enable more adaptive modeling approaches and yield more precise clinical decision rules.

The second most impactful limitation is the exclusion of clinical health indicators, including physical mobility levels, mental health diagnoses such as PTSD and depression, pain severity ratings, and continuous cognitive severity scores beyond the binary eligibility threshold applied in this study. These variables are strongly theoretically linked to recreational therapy engagement, and their absence limits both the explanatory completeness of the predictive framework and its clinical utility for identifying the specific health-related mechanisms driving disengagement. Their exclusion was necessitated by the survey-based data collection design, which was intentionally scoped to variables obtainable through routine recreational therapy assessments without requiring electronic health record linkage. Future studies should prioritize EHR integration to incorporate these indicators as predictors, which would substantially improve model comprehensiveness and provide a more complete clinical picture of veteran disengagement risk.

The third most impactful limitation is the single-site cross-sectional design, which restricts generalizability to other veteran long-term care facilities with different organizational cultures, staffing models, or population characteristics. The predictive models were developed and validated exclusively at NYSVETS Home at Oxford, and their performance in other settings, including VA-affiliated facilities, privately operated veteran homes, or facilities serving more demographically diverse veteran populations, remains unknown. Multi-site prospective studies are needed to establish the external validity of these findings.

Fourth, reliance on self-reported participation measures introduces potential bias related to overreporting, recall errors, and social desirability effects, which could systematically distort both the outcome variables and several predictor measures. Objective participation tracking systems, such as electronic attendance records or wearable activity monitors, would provide more accurate and bias-resistant outcome data in future investigations.

Fifth, the survey instrument was newly developed for this investigation and has not undergone formal psychometric evaluation. While expert review by licensed recreational therapists and pilot testing with a subset of residents helped ensure content relevance and clarity, formal statistical assessments of internal consistency (e.g., Cronbach's alpha), test-retest reliability, and construct validity through factor analysis were not conducted due to sample size constraints. Future studies should incorporate full psychometric validation of the instrument before broader deployment.

Finally, the conservative hyperparameter choices that were necessary to prevent overfitting in this small-sample context may have introduced a degree of underfitting, potentially limiting model performance below what might be achievable with larger datasets and more adaptive tuning strategies. This trade-off is inherent to the bias-variance considerations of small-sample machine learning and represents a modeling constraint rather than an analytical error, but it suggests that reported performance metrics should be interpreted as conservative estimates of true model capability.

### 4.4 Practical challenges and implementation considerations

Beyond the methodological limitations discussed above, several practical challenges encountered during data collection and anticipated barriers to model deployment merit explicit discussion for clinical audiences considering similar research or implementation efforts.

#### 4.4.1 Data collection challenges

Conducting research within a long-term care facility serving an older veteran population presented several logistical and ethical challenges that directly shaped the study design and sample characteristics. Cognitive eligibility screening using the Brief Interview for Mental Status (BIMS) was a necessary ethical safeguard but reduced the eligible pool from approximately 120 residents to 76, constraining the achievable sample size. Among eligible residents, survey completion required individualized staff assistance in several cases to accommodate physical limitations such as reduced dexterity or visual impairment, increasing the time burden on recreational therapy staff during active programming hours. The electronic administration via Qualtrics, while ensuring HIPAA compliance, presented usability challenges for some older veterans with limited technology familiarity, necessitating one-on-one support from research staff to ensure response accuracy without introducing interviewer bias. These recruitment and administration realities are important context for interpreting the final sample size of 57 and should be anticipated by researchers designing similar studies in veteran long-term care settings. Additionally, the self-administered survey format, while preserving participant autonomy, introduced variability in how residents interpreted participation frequency categories, particularly the distinction between low and moderate participation levels. Future implementations should consider structured interview formats or objective attendance record linkage to reduce response ambiguity and improve measurement precision.

#### 4.4.2 Model deployment challenges

Translating the predictive models developed in this study into routine clinical practice presents several implementation challenges that should be acknowledged transparently. First, the clinical decision rules derived from the interpretable decision tree require staff to collect and document specific data points, including activity preference diversity counts and standardized satisfaction scores, during routine assessments. While these variables are readily obtainable through brief structured interviews, their systematic collection represents an additional workflow step that must be integrated into existing admission and care planning processes without creating undue burden for recreational therapy staff. Second, the models were developed using a structured survey instrument specifically designed for this investigation, meaning that deployment in other facilities would require either adoption of the same survey tool or careful mapping of existing assessment instruments to the predictor variables used in the models. Facilities using different activity preference or satisfaction measurement approaches may obtain different predictor distributions, potentially affecting model calibration and clinical rule applicability. Third, successful implementation of prediction-guided intervention targeting requires organizational readiness, including staff training in interpreting model outputs, institutional support for early intervention protocols, and clear pathways for translating risk scores into actionable care plan modifications. Without these structural supports, even well-validated predictive models risk becoming unused additions to clinical documentation rather than tools that meaningfully change care delivery. Future implementation research should prioritize workflow integration studies, staff training evaluations, and prospective assessments of whether prediction-guided targeting produces measurable improvements in RT engagement rates relative to standard clinical judgment alone.

## 5 Conclusions

Veterans in long-term care facilities represent a rapidly growing population with unique therapeutic needs, yet engagement in recreational therapy programs varies considerably among residents. This study employed machine learning methodologies to develop predictive models for recreational therapy engagement among 57 veterans at a specialized long-term care facility. Data were collected through a comprehensive survey capturing demographic characteristics, participation patterns, and activity preferences. Five machine learning algorithms were evaluated using cross-validation approaches tailored to address class imbalance challenges. The Random Forest algorithm achieved optimal performance with F1-scores of 0.860 for high participation prediction and balanced accuracy of 0.619 for any participation prediction. Activity preference diversity and facility tenure emerged as primary predictors, with veterans demonstrating interest in more than 4.5 activity categories showing substantially higher participation rates. The analysis revealed a critical 1.5-year adaptation period during which new residents are most vulnerable to disengagement, while veterans with high preference diversity and satisfaction scores above 3.84 achieved 100% observed probability of sustained participation [95% exact binomial CI: (47.8%, 100%); *n* = 5], a finding that should be prospectively validated in larger samples.

These findings should be interpreted within the scope of a rigorously conducted single-site pilot investigation. The predictive models developed here are not yet ready for broad clinical deployment but provide a validated methodological framework and preliminary evidence base to support larger-scale prospective studies. The decision rules and risk identification criteria generated offer promising directions for optimizing recreational therapy delivery through systematic early intervention targeting, pending external validation across diverse veteran long-term care settings.

## Data Availability

The raw data supporting the conclusions of this article will be made available by the authors, without undue reservation.
