# Supplementary material for: Predicting recreational therapy engagement in veterans’ long-term care: a machine learning approach
Source: Front Health Serv. 2026 Apr 30;6:1735411. doi: 10.3389/frhs.2026.1735411 (PMC13171575; doi:10.3389/frhs.2026.1735411)
Supplement: Supplementary file 1 [file Datasheet1.pdf]

Supplementary Tables: Feature Engineering Details

Supplementary Table S1. Demographic Feature Engineering

Original Variable	Original Values	Engineered Feature	Encoding Method	Encoded Values
**Q1 (Age)**	"31-40", "41-50", "51-60", "61-70", "71-80", "81-90", "91 and above"	Age_Numeric	Ordinal numeric	0=Under 70, 1=71-80, 2=81-90, 3=91+
**Q2 (Gender)**	"Male", "Female", "Other", "Prefer not to say"	Gender_Male	Binary indicator	1=Male, 0=Female/Other
**Q3 (Residency)**	"Less than 6 months", "6 months to 1 year", "1-2 years", "More than 2 years"	Residency_Numeric	Ordinal numeric	0=New (<1 year), 1=Established (1-2 years), 2=Long-term (>2 years)

Supplementary Table S2. Activity Preference Feature Engineering

Survey Question	Activity Type	Engineered Feature	Encoding Method	Values	Sample Size (n)	Selection Rate (%)
**Q9_1**	Music Therapy	Music_Therapy_Selected	Binary indicator	1=Selected, 0=Not selected	22	38.6
**Q9_2**	Physical Exercise	Physical_Exercise_Selected	Binary indicator	1=Selected, 0=Not selected	30	52.6
**Q9_3**	Group Activities	Group_Activities_Selected	Binary indicator	1=Selected, 0=Not selected	25	43.9
**Q9_4**	Art Therapy	Art_Therapy_Selected	Binary indicator	1=Selected, 0=Not selected	14	24.6
**Q9_5**	Gardening	Gardening_Selected	Binary indicator	1=Selected, 0=Not selected	14	24.6
**Q9_6**	Mind Exercises	Mind_Exercises_Selected	Binary indicator	1=Selected, 0=Not selected	30	52.6

**Q9_9**	Spiritual Activities	Spiritual_Activities_Selected	Binary indicator	1=Selected, 0=Not selected	16	28.1
**Q9_8**	Digital Activities	Digital_Activities_Selected	Binary indicator	1=Selected, 0=Not selected	7	12.3

Supplementary Table S3. Composite Feature Engineering

Composite Feature	Calculation Method	Component Features	Range	Mean ± SD
**Total_Preferences**	Sum of all activity selections	Sum of all 8 activity binary indicators	0-8	2.73 ± 1.89
**Preference_Diversity**	Count of distinct categories	Count of unique activity categories selected	0-8	2.73 ± 1.89
**Physical_Preference**	Maximum of physical activities	Max(Physical_Exercise_Selected)	0-1	0.53 ± 0.50
**Social_Preference**	Maximum of social activities	Max(Group_Activities_Selected, Music_Therapy_Selected)	0-1	0.65 ± 0.48
**Creative_Preference**	Maximum of creative activities	Max(Art_Therapy_Selected, Gardening_Selected)	0-1	0.42 ± 0.50
**Spiritual_Mental_Preference**	Maximum of cognitive/spiritual activities	Max(Spiritual_Activities_Selected, Mind_Exercises_Selected)	0-1	0.65 ± 0.48

Supplementary Table S4. Satisfaction Feature Engineering

Original Variable	Original Values	Engineered Feature	Transformation	Final Values	Missing Data Handling
**Q6 (RT Satisfaction)**	"Very dissatisfied", "Dissatisfied", "Neutral", "Satisfied",	Satisfaction_Score_Filled	Likert scale mapping	1=Very dissatisfied to 5=Very satisfied	Mean imputation (3.68) for missing

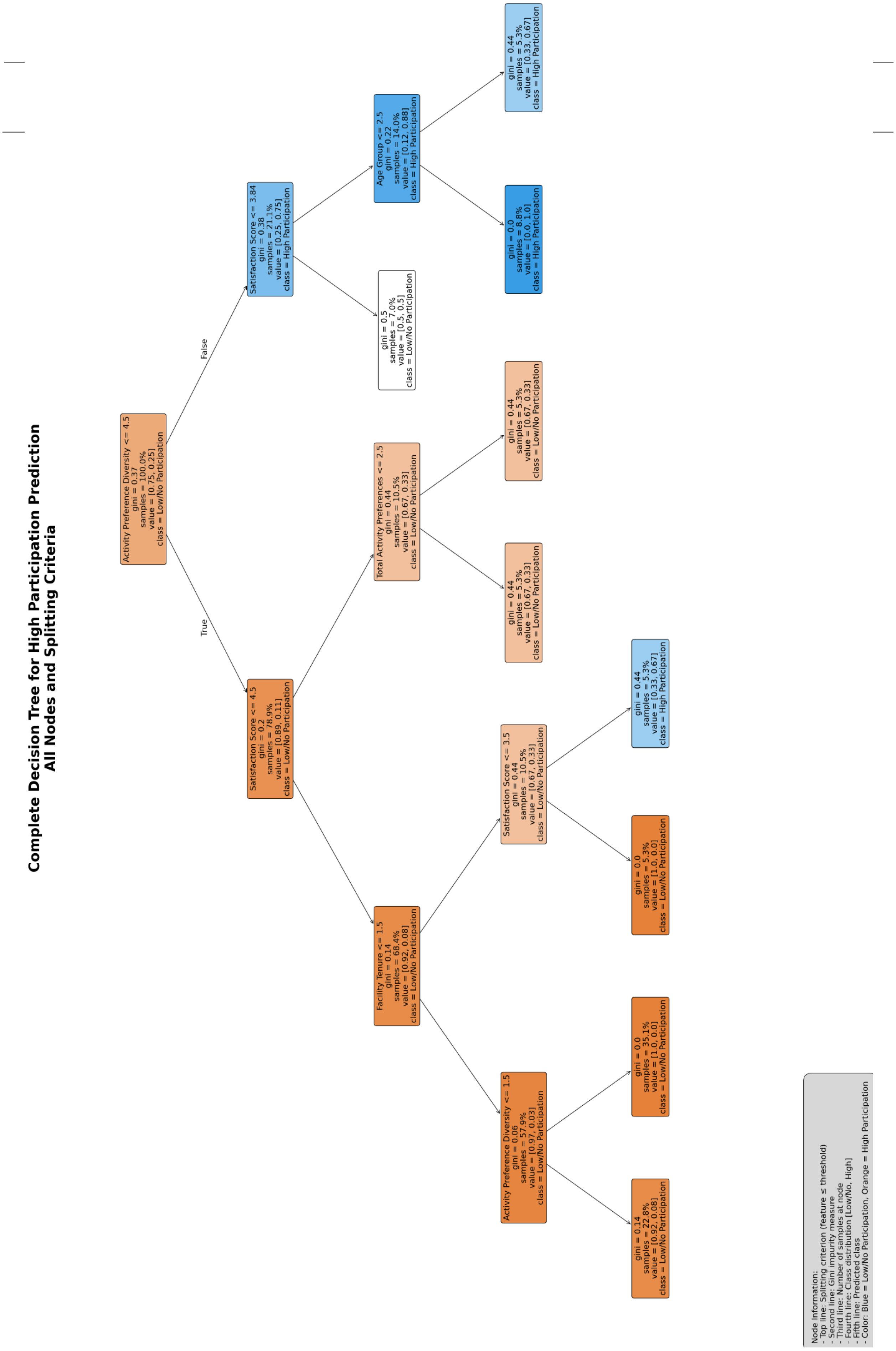

Figure S1*: Complete Decision Tree Diagram for High Participation Prediction in Recreational Therapy*

Supplementary Table S5. Comparison of Feature Sets: Statistical Optimization vs. Clinical Implementation.

Aspect	Random Forest Models (Statistical Optimization)	Decision Tree Rules (Clinical Implementation)
**Primary Purpose**	Maximum predictive accuracy for research insights	Practical clinical screening and intervention targeting
**Feature Selection Method**	SelectKBest with f_classif scoring (statistical)	Clinical accessibility and universal availability
**Number of Features**	6 features (sample-to-variable ratio optimized)	6-8 features (interpretability optimized)
**Selected Features**	Preference Diversity, Total Preferences, Group Activities Selected, Spiritual Activities Selected, Residency Numeric, Social Preference	Age Numeric, Gender Male, Residency Numeric, Total Preferences, Preference Diversity, Satisfaction Score Filled
**Assessment Requirements**	Detailed activity preference survey across 8 specific categories	Basic demographic data plus simple preference count and satisfaction rating
**Clinical Feasibility**	Requires comprehensive survey administration	Available during routine clinical assessments
**Generalizability**	Facility-specific, depends on available programs	Universal across different care settings
**Implementation Burden**	High, requires 10-15 minute survey	Low, requires 2-3 minute assessment
**Primary Output**	Probability estimates for research and quality improvement	Explicit if-then rules for clinical decision-making
**Staff Training Required**	Moderate, survey administration and interpretation	Minimal, simple screening criteria
**Validation Approach**	LOOCV with F1-score optimization	Interpretability and clinical utility focus
**Target End-Users**	Researchers, quality improvement teams	Frontline recreational therapy staff

Supplementary Table S6. Rankings of predictors across multiple feature selection methods, including ANOVA F-Score, Mutual Information, Random Forest, and Decision Tree importance measures, along with stability scores representing the percentage of cross-validation folds in which each feature was selected. Higher stability scores indicate stronger consensus across methods and folds.

Feature	ANOVA F-Score Rank	Mutual Information Rank	Random Forest Importance Rank	Decision Tree Importance Rank	Stability Score (%)
Preference Diversity	1	1	1	1	95
Total Preferences	2	2	2	3	92
Residency Length	3	3	4	2	88
Group Activities	4	5	3	4	76
Spiritual Activities	5	4	5	5	74
Physical Exercise	6	6	6	7	70
Music Therapy	7	7	8	6	68
Art Therapy	8	9	9	9	65
Gardening	9	8	7	8	60
