# Supplementary material for: Predicting recreational therapy engagement in veterans’ long-term care: a machine learning approach
Source: Front Health Serv. 2026 Apr 30;6:1735411. doi: 10.3389/frhs.2026.1735411 (PMC13171575; doi:10.3389/frhs.2026.1735411)
Supplement: Supplementary file 2 [file Datasheet2.pdf]

SF2.Model Stability and Statistical Validation for High Participation Prediction: Permutation Test and Bootstrap Confidence Interval Analysis (Random Forest, n = 57)

Panel A. Summary of Observed Model Performance

Metric	Value	Description
**Observed LOOCV F1-score**	0.860	Mean F1 across all 57 LOOCV folds
**Standard deviation (SD)**	0.347	Per-fold bimodal variance; reflects LOOCV binary outcome structure
**Standard error of the mean (SE)**	0.046	SE = SD / √n = 0.347 / √57
**95% CI (SE-based)**	[0.770, 0.950]	Mean ± 1.96 × SE; precision of the mean performance estimate
**Sample size (n)**	57	High participators: 14 (24.6%); non-high: 43 (75.4%)

Panel B. Permutation Test Results (1,000 Iterations)

Parameter	Value	Interpretation
**Number of permutations**	1,000	Random label shuffles preserving class distribution
**Null distribution mean F1**	0.381	Average F1 under random label assignment (chance level)
**Null distribution SD**	0.163	Variability of F1 across permuted datasets
**Null distribution maximum F1**	0.783	Highest F1 observed across all 1,000 permutations
**Null distribution 95th percentile**	0.671	Upper bound of expected chance performance
**Observed F1 vs. null maximum**	0.860 > 0.783	Observed performance exceeds maximum chance-level F1
**Permutations ≥ observed F1**	18 / 1,000	Number of permuted F1 values meeting or exceeding 0.860
**Permutation p-value**	0.018	Proportion of permutations ≥ observed F1; statistically significant ($p < 0.05$)

Panel C. Bootstrap Confidence Interval Results (500 Stratified Resamples)

Parameter	Value	Interpretation
**Number of bootstrap resamples**	500	Stratified resamples preserving 24.6% / 75.4% class split
**Valid resamples used**	487 / 500	13 resamples excluded due to single-class representation
**Bootstrap mean F1**	0.853	Mean F1 across bootstrap resamples; consistent with observed 0.860
**Bootstrap SD**	0.058	Variability of F1 across resampled datasets
**2.5th percentile**	0.741	Lower bound of bootstrap distribution
**97.5th percentile**	0.947	Upper bound of bootstrap distribution
**95% Bootstrap CI (percentile method)**	[0.741, 0.947]	Assumption-independent stability estimate; consistent with SE-based CI [0.770, 0.950]

Panel D. Convergent Evidence Summary

Stability indicator	Result	Threshold	Assessment
**SE-based 95% CI width**	0.180	< 0.30	Stable
**Permutation p-value**	0.018	< 0.05	Significant
**Bootstrap 95% CI lower bound**	0.741	> 0.70	Acceptable
**Overfitting gap (training vs. validation)**	0.030	< 0.15	Stable
**Consistency: SE-CI vs. Bootstrap-CI**	Overlap > 95%	Substantial overlap	Consistent

Notes. LOOCV = Leave-One-Out Cross-Validation; SE = standard error of the mean; CI = confidence interval. The relatively large standard deviation across LOOCV folds reflects variability inherent to cross-validation with small datasets, where each fold contains a single observation in the test set. The standard error of the mean (SE = 0.046) provides the appropriate measure of precision for the mean performance estimate. Permutation test p-values were calculated as the proportion of permuted F1 scores meeting or exceeding the observed value (0.860) across 1,000 label permutations. Bootstrap confidence intervals were computed using the percentile method from 500 stratified resamples.
